# Katanin p60 Contributes to Microtubule Instability around the Midbody and Facilitates Cytokinesis in Rat Cells

**DOI:** 10.1371/journal.pone.0080392

**Published:** 2013-11-26

**Authors:** Moe Matsuo, Tetsuhiro Shimodaira, Takashi Kasama, Yukie Hata, Ayumi Echigo, Masaki Okabe, Kazuya Arai, Yasutaka Makino, Shin-Ichiro Niwa, Hideyuki Saya, Toshihiko Kishimoto

**Affiliations:** 1 Department of Biomolecular Science, Faculty of Science, Toho University, Funabashi, Chiba, Japan; 2 Link Genomics Co., Ltd., Tokyo, Japan; 3 Division of Gene Regulation, Institute for Advanced Medical Research, School of Medicine, Keio University, Tokyo, Japan; 4 Proteome Analysis Center, Faculty of Science, Toho University, Funabashi, Chiba, Japan; University of Connecticut, Storrs, United States of America

## Abstract

The completion of cytokinesis is crucial for mitotic cell division. Cleavage furrow ingression is followed by the breaking and resealing of the intercellular bridge, but the detailed mechanism underlying this phenomenon remains unknown. Katanin is a microtubule-severing protein comprised of an AAA ATPase subunit and an accessory subunit designated as p60 and p80, respectively. Localization of katanin p60 was observed at the midzone to midbody from anaphase to cytokinesis in rat cells, and showed a ring-shaped distribution in the gap between the inside of the contractile ring and the central spindle bundle in telophase. Katanin p60 did not bind with p80 at the midzone or midbody, and localization was shown to be dependent on microtubules. At the central spindle and the midbody, no microtubule growth plus termini were seen with katanin p60, and microtubule density was inversely correlated with katanin p60 density in the region of katanin p60 localization that seemed to lead to microtubule destabilization at the midbody. Inhibition of katanin p60 resulted in incomplete cytokinesis by regression and thus caused the appearance of binucleate cells. These results suggest that katanin p60 contributes to microtubule instability at the midzone and midbody and facilitates cytokinesis in rat cells.

## Introduction

Cytokinesis, the division of the cytoplasm, is a critical step in the cell division cycle for the formation of two separate genetically identical daughter cells. To avoid the generation of aneuploid daughter cells, cytokinesis must be subject to spatial and temporal controls [1]. Failure of cytokinesis leads to progressive genomic instability and tumorigenesis [2]. Cytokinesis of animal cells is initiated during anaphase, when the mitotic spindle reorganizes to form the central spindle, a dense array of antiparallel microtubules midway between the two centrosomal asters. Together with microtubules from the spindle asters, the central spindle defines the position of the division plane between the segregated chromosomes. This spatial signal is transmitted through a pathway involving the small GTPase RhoA, leading to the assembly of an actomyosin ring at the equatorial cell cortex. Contraction of the actomyosin ring pulls the overlying plasma membrane toward the center of the cell where it reaches the central spindle. The central spindle is essential for completion of cytokinesis in animal cells [3-6]. 

Central spindle assembly begins in early anaphase when nonkinetochore spindle microtubules become bundled. The microtubule bundles of the central spindle span the interpolar region of the anaphase spindle, and there is a narrow region of overlap between the two sets of antiparallel microtubules [7]. These bundles become condensed during cytokinesis and eventually develop into the cell midbody that consists of tightly packed microtubules and associated proteins [3,8]. From anaphase to cytokinesis, the dynamics of the central spindle show dramatic regulation, but the underlying mechanisms remain unclear. 

Microtubule synthesis during mitosis has been thought to occur mainly at the centrosome (spindle pole); however, noncentrosomal microtubule synthesis for spindle organization has recently been reported [9-11]. The chromatin-dependent microtubule generation pathway has been investigated in detail, and the results indicated that Ran-GTP and the chromosome passenger complex, which are enriched around the chromosomes, induce microtubule nucleation [9-11]. In addition, the microtubule-dependent microtubule generation pathway, which was originally identified in the cells of fission yeast [12] and higher plants [13], involves the generation of microtubules throughout the spindle and not necessarily near the chromosomes [14-16].

Augmin, a protein complex recently identified in *Drosophila*, is a critical factor for centrosome-independent, spindle-based microtubule generation [15-17]. The augmin-dependent microtubule generation pathway is thought to be important for formation and maintenance of the central spindle. However, the mechanisms of microtubule destabilization and breakdown in mitosis, especially in cytokinesis, are still unknown.

Katanin, spastin, and fidgetin are members of a family of AAA ATPases (ATPase Associated with various cellular Activities) that influence microtubule dynamics in a variety of organisms [18,19]. These enzymes sever microtubules along their length, thus shortening them and increasing the overall number of microtubules as well as increasing the pool of free tubulin molecules, which can nucleate new microtubules. It was reported that spastin, a microtubule-severing protein that is associated with the ESCRT (endosomal sorting complex required for transport) complex machinery, couples severing of microtubules to membrane traffic in completion of cytokinesis, especially affecting elongation of the tubules connecting pairs of daughter cells and loss of midbody microtubules [20,21]. Spastin knockdown has also been reported to result in disorganization of the central spindle microtubules and defective delivery of endosomes to the intracellular bridge [22]. Thus, there is a correlation between central spindle microtubule dynamics and microtubule severing, but the contributions of other microtubule-severing proteins for cytokinesis and the significance of the severing or depolymerization of microtubules remains unclear.

Katanin is a heterodimeric protein consisting of an AAA ATPase subunit and an accessory subunit designated as p60 and p80, respectively. The ATPase subunit p60 alone can sever microtubules, but this activity is enhanced by the p80 subunit [23,24]. The microtubule-severing activity of katanin and its conserved localization at the spindle poles suggest that it plays a role in regulating spindle stability [25,26]. In proliferating cells, katanin contributes to mitosis by severing the microtubules at the mitotic spindle poles and increasing the number of minus-ends. Moreover, katanin located at the spindle poles also elicits severing activity to shorten the metaphase spindles. The function of katanin on the centrosome has been characterized in detail, but recent studies have indicated multiple cellular locations and functions of katanin [22,27]. In *Drosophila*, katanin was shown to be localized on anaphase chromosomes where it stimulates microtubule plus-end depolymerization and Pacman-based chromatid motility [18]. In rat cells, the pluripotent tumor suppressor LAPSER1/LZTS2 binds the katanin p80 subunit directly and shares centrosomal and midbody localization with the p80 subunit, and LAPSER1 has been shown to inhibit the microtubule-severing activity of katanin by binding to the p80 subunit [28,29]. These studies suggested a function of katanin not only at the centrosome but also at the chromosome and midbody. However, there have been few reports regarding the relationship between katanin p60 and the midbody. Recently, the solution structure of the katanin p60 N-terminal domain was determined by nuclear magnetic resonance spectroscopy (NMR), and was shown to have striking similarity to microtubule interacting and trafficking (MIT) domains, which adopt an antiparallel three-stranded helix bundle conformation [30]. Moreover, helices 2 and 3 of the katanin p60 MIT domain were well conserved with the MIT domain of Vps4, which is a homologous protein that promotes disassembly of the ESCRT-III membrane skeleton complex [30]. Recently, it was reported that human ESCRT-III and VPS4 proteins are important for centrosome and spindle maintenance [31]. These studies suggested functional and structural relationships between spindle and central spindle dynamics, including katanin p60. Recent RNA interference (RNAi) experiments indicated that cytokinesis in bloodstream-stage *Trypanosoma brucei* requires a family of katanins and spastin [23]. Moreover, katanin was shown to be important for plant cell division [32]. However, although the function of katanin p60 at the spindle pole has been analyzed in detail, its other cellular functions remain unknown.

Here, we report the novel localization and microtubule destabilization function of katanin p60 at the midzone and the midbody during cell division. Finally, we show that midzone katanin p60 plays an important role in facilitating the completion of cytokinesis.

## Materials and Methods

### Cell lines & Cell culture

The rat fibroblast cell line 3Y1, rat liver cell line RL34, and rat hepatocellular carcinoma cell line FAA-HTC1 were obtained from Health Science Research Resources (Osaka, Japan). The rat fibroblast 3Y1 cell line and rat liver cell line RL34 were maintained in Dulbecco’s Modified Eagle’s Medium (DMEM; Sigma) supplemented with 10% fetal bovine serum (FCS). The rat hepatocellular carcinoma cell line FAA-HTC1 was maintained in William’s *E medium* supplemented with 10% FCS and l-glutamine (Invitrogen). For immunofluorescence analyses, cells were cultured on cover glasses, and with flexiPERM (Sigma) for small interfering RNA (siRNA) analyses.

### Antibodies and reagents

Mouse monoclonal antibodies against β-tubulin (Sigma), actin (Sigma), and EB1 (Becton Dickinson) were used. Anti-EB1 antibody reactivity was checked by double staining with rat monoclonal anti-α-tubulin antibody (Santa Cruz) (Figure S1). Chicken polyclonal antibody against p80 katanin was obtained from GeneWay Biotech and Sigma. Nocodazole, paclitaxel, and blebbistatin were purchased from Sigma.

### Expression of recombinant rat katanin p60 protein and generation of anti-katanin p60 antibody

First, the rat katanin p60 subunit was cloned from a rat liver cDNA library by PCR. Cloned katanin p60 cDNA encoded a polypeptide of 491 amino acids with a predicted molecular weight of 55 kDa, and its nucleotide sequence showed a complete match to that in the NCBI database (katanin p60 (ATPase-containing) subunit A1 [*Rattus norvegicus*]; GenBank accession number BC097929.1). Recombinant rat katanin p60 was expressed by the pET system in BL21 (DE3) pLysS. Expressed recombinant katanin p60 was purified with Ni-NTA agarose (Qiagen) under denaturing conditions, followed by preparative sodium dodecyl sulfate-polyacrylamide gel electrophoresis (SDS-PAGE; gel thickness, 5 mm, SDS 0.1 %), and the katanin-containing band was finally electroeluted. Purified recombinant katanin p60 had > 95% purity.

Anti-rat katanin p60 antiserum was generated by immunization of rabbits with recombinant protein with and without hapten. After immunization, antiserum was purified using an affinity column with immobilized recombinant katanin p60 as the antigen on NHS-activated Sepharose (GE Healthcare). On Western blotting analyses, affinity-purified anti-katanin p60 antibodies could detect endogenous rat katanin p60 specifically from 3Y1 cell extract (Figure S2 A), and reacted specifically with katanin p60 and not with recombinant rat KATNAL1, which is the closest paralog of katanin p60 (Figure S2 B).

### RNA interference

RNA interference for rat katanin p60 was performed using SMART pool siRNAs (Dharmacon; 5'-GGGAGGAGCUAUUACGAAUUU-3', 5'-GCUGUUCGUUGUCGUGAAAUU-3', 5'-GGAUCAUGCUAACUCGAGAUU-3', and 5'-CAUUGAAAGAUACGAGAAAUU-3') and each siRNA independently. ON-TARGET plus siCONTROL non-target siRNA (Dharmacon) was used as control siRNA. siRNAs were transfected using Lipofectamine RNAi MAX (Invitrogen).

### Immunocytochemistry

Indirect immunostaining of cell lines was performed as follows. Cells were grown on cover glasses followed by fixation in methanol with 1 mM ethylene glycol tetraacetic acid (EGTA) for 3 min at -20°C, rinsing for 5 min twice with phosphate buffered saline containing Tween 20 (PBST) and once with phosphate buffered saline (PBS), blocking with 1.5% BSA in PBST for 30 min at room temperature, and incubation with primary antibodies for 1 h at room temperature. After further washing with PBST, cells were stained with secondary antibodies (Molecular Probes) for 30 min. After washing with PBS, cells were stained with 0.2 mg/mL of 4',6-diamidino-2-phenylindole (DAPI) for 3 min. Fluorescent signals were detected using an Axioskop II fluorescence microscope (Carl Zeiss) and FV1000-D confocal microscope (Olympus).

### Immunofluorescence analysis

Fluorescence signals detected by confocal microscopy were analyzed using Fluoview viewer software (Olympus). Regions of interest (ROI) were selected manually and fluorescence was measured. After subtraction of background fluorescence, the net fluorescence of each ROI was determined.

### Time-lapse analysis

A total of 10^3^ 3Y1 cells were cultured in each well of 96-well plates and incubated at 37°C under 5% CO_2_ for 16 h. Then, 10 µl of 2 µM siRNA (control or katanin p60) was mixed with 8 µl of Opti-MEM (Invitrogen), and then mixed with 2 µl of Lipofectamine RNAi MAX and Opti-MEM mixture (1:4). Cells were washed and 80 µl/well of Opti-MEM was added. Then, 20 µl/well of siRNA mixture was added and incubated 4 h at 37°C. Transfection medium was exchanged for 150 µl/well DMEM (Sigma) supplemented with 10% FBS (Hyclone). Five hours after transfection, time-lapse analysis was started and performed for 72 h. Time-lapse analysis was performed using a TE2000 microscope (Nikon), microscope incubation system with GM2000 CO_2_ regulatory apparatus (Tokai Hit), and CoolSnapK4 CCD camera (Roper Scientific). Time-lapse imaging data were analyzed by MetaMorph Imaging software (Universal Imaging).

## Results

### Rat katanin p60 is localized to both the spindle pole and midbody during mitosis

**Figure 1:**
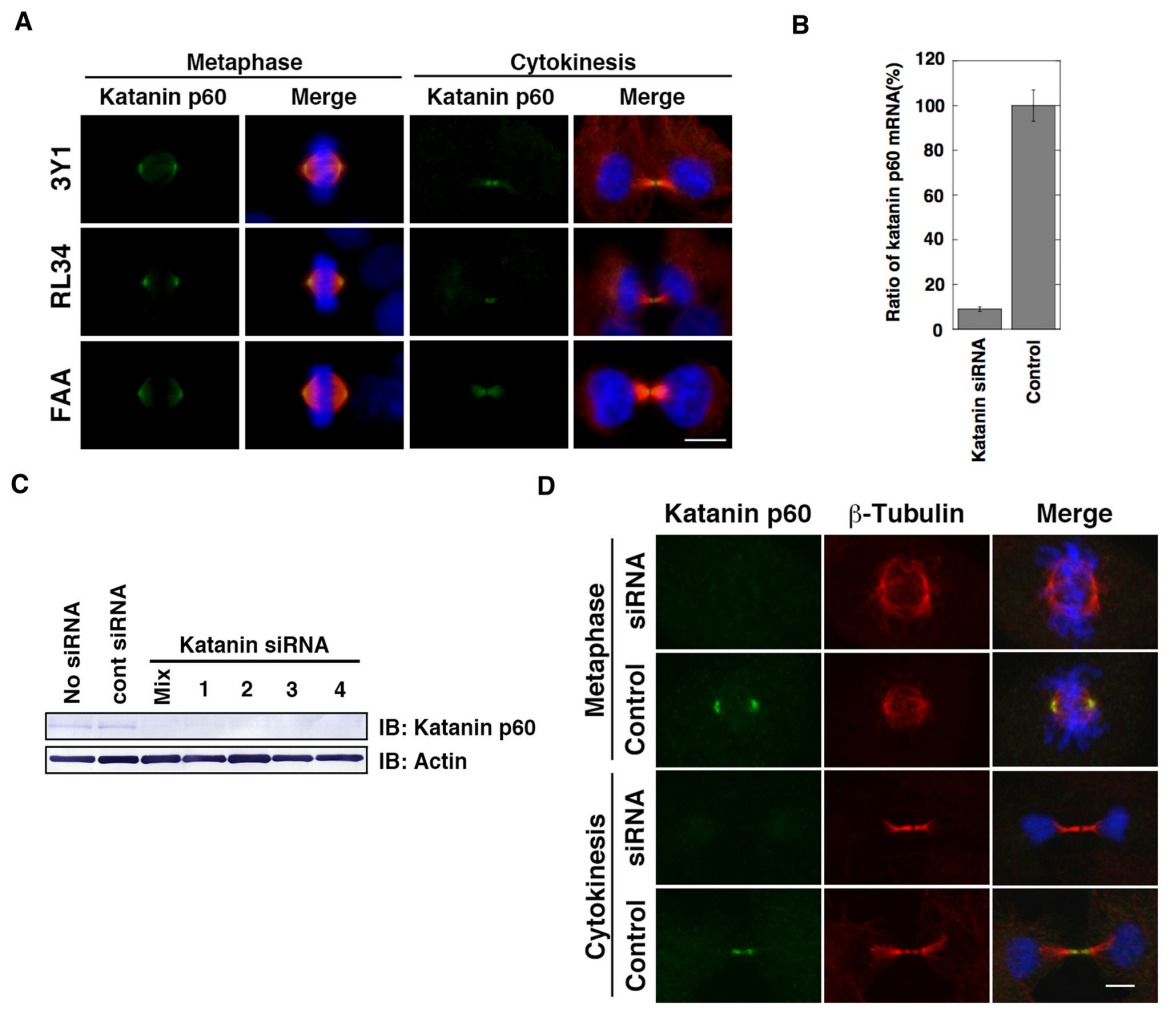
Katanin p60 is localized at both the spindle pole and midbody. A. 3Y1, RL34, and FAA indicate immunostained images of 3Y1 cells, RL34 cells, and FAA-HTC1 cells, respectively. Cells were labeled for katanin p60 (green), β-tubulin (microtubules; red), and DNA (blue). Merge indicates merged images of katanin p60, ß-tubulin, and DNA, showing the localization of katanin p60 at the spindle pole (Metaphase) and midbody (Cytokinesis) during mitosis. Scale bars: 10 µm. Samples were fixed in methanol and analyzed by fluorescence microscopy (Axioskop II; Carl Zeiss). B. Inhibition of katanin p60 by katanin siRNA was determined by quantitative RT-PCR 24 h after transfection. Katanin p60 mRNA expression levels were normalized relative to TATA Binding Protein (TBP) mRNA as an internal control. C. Inhibition of katanin p60 by katanin siRNA was determined by Western blotting analysis 48 h after transfection. Four different siRNAs derived from the rat katanin p60 sequence were used independently (1 - 4) and mixed (Mix). IB indicates immunoblotting with anti-katanin p60 antibody (IB: Katanin p60) and with anti-actin antibody (IB: Actin). D. 3Y1 cells labeled for katanin p60 (green), β-tubulin (red), and DNA (blue). Merge indicates merged images of katanin p60, β-tubulin, and DNA, showing the localization of katanin p60 at the spindle pole (Metaphase) and midbody (Cytokinesis) during mitosis. 3Y1 cells were transfected with control siRNA (Control) or katanin p60 siRNA (siRNA). Scale bars: 10 µm. Samples were fixed in methanol and analyzed by confocal laser scanning fluorescence microscopy (FV-1000D; Olympus).

To examine the function of microtubule severing during mitosis, we selected katanin p60 as a key factor in the severing of microtubules and rat cell lines. First, we performed indirect immunostaining (Figure 1A) with affinity-purified anti-katanin p60 antibody (the specificity of the antibody was determined by Western blotting, as shown in Figure S2) to identify the precise localization of katanin p60 during the rat cell cycle. Interestingly, katanin p60 was clearly localized at both the spindle pole and midbody during mitosis in rat 3Y1 cells (fibroblast cell line; Figure 1A 3Y1), RL34 cells (normal liver cell line; Figure 1A RL34), and FAA-HTC1 cells (hepatoma cell line; Figure 1A FAA). These data indicated that rat katanin p60 is localized not only at the spindle pole but also at the midbody. Antibody preabsorbed with recombinant katanin p60 did not detect localization of katanin (Figure S3) suggesting that the observed midbody localization of katanin p60 was not an artifact. Furthermore, to determine the specific localization of katanin p60, we performed inhibition experiments using katanin siRNA (Figure 1B - D). The extent of inhibition of katanin p60 expression was confirmed by quantitative reverse transcription polymerase chain reaction (qRT-PCR) and Western blotting analyses. Katanin p60 mRNA level decreased by > 90% (Figure 1B), and katanin p60 protein expression was mostly repressed by katanin siRNA transfection (Figure 1C). To determine whether katanin p60 midbody localization was affected by siRNA treatment, the localization of katanin p60 was examined in siRNA-treated cells. The intensities of signals indicating katanin p60 localization at both the spindle pole and midbody were markedly reduced (Figure 1D siRNA). Midbody katanin p60 was more difficult to repress by siRNA treatment, and a clear reduction in expression level was observed only by repeating the siRNA treatment twice (Figure 1D Cytokinesis). These results indicated that midbody localization of katanin was equally plausible to localization at the spindle pole, and that katanin p60 may have significant roles at both the midbody and spindle pole.

### Midbody localization of katanin is dependent on central spindle structure

**Figure 2:**
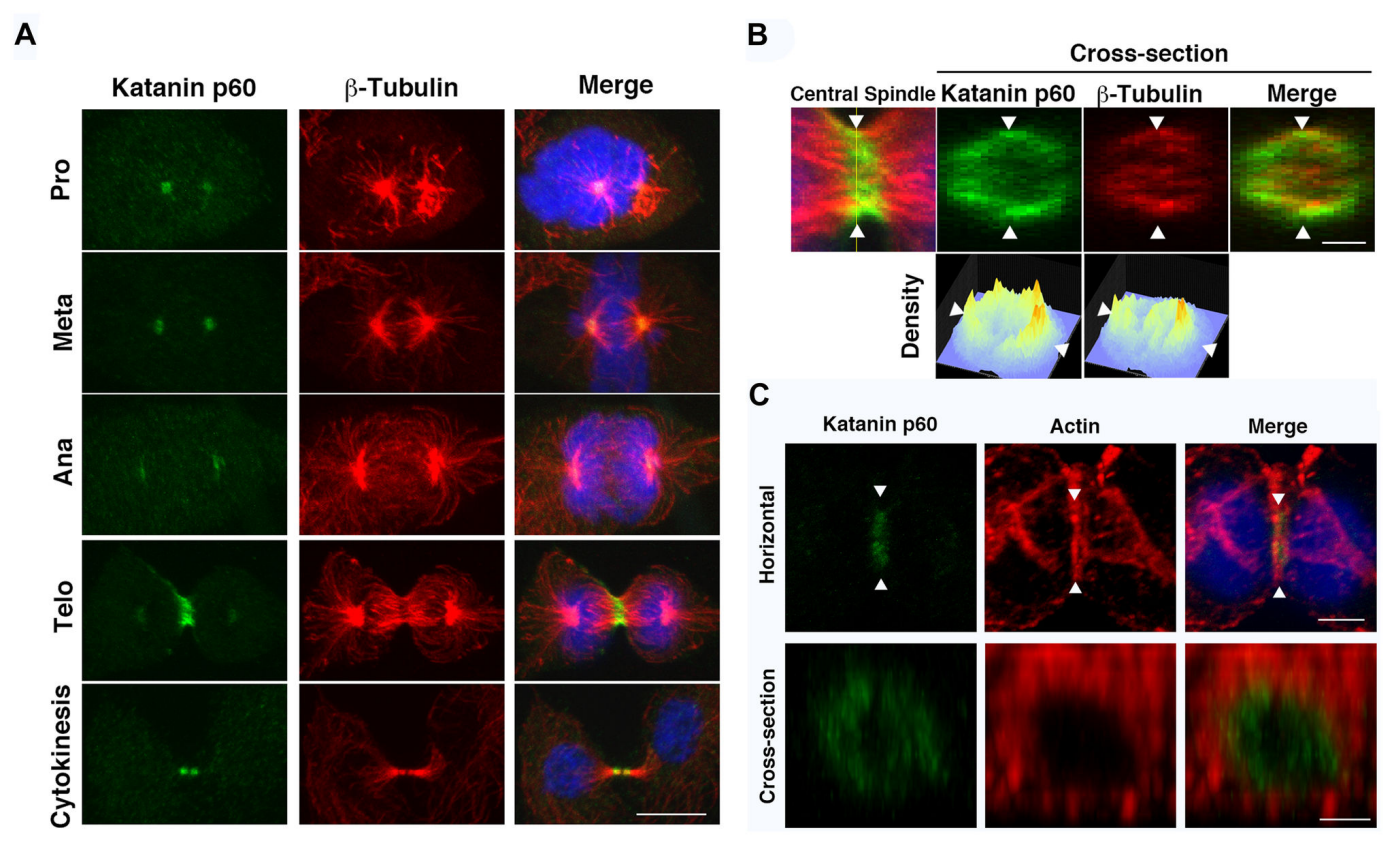
Subcellular localization of endogenous katanin p60. A. 3Y1 cells labeled for katanin p60 (green), β-tubulin (red), and DNA (blue). Merge indicates merged images of katanin p60, β-tubulin, and DNA, showing the localization of katanin p60 during mitosis. Pro, Meta, Ana, Telo, and Cytokinesis indicate images of prophase, metaphase, anaphase, telophase, and cytokinesis, respectively. Scale bars: 10 µm. Samples were fixed in methanol and analyzed by confocal laser scanning fluorescence microscopy (FV-1000D; Olympus). B. Higher magnification micrograph around the central spindle in Figure 2. Telophase Merged image is shown in the upper left (Central spindle). Vertical analysis images of the distribution of katanin p60 (green), β-tubulin (red), and DNA (blue). Merge indicates merged images of katanin p60, β-tubulin, and DNA, showing a cross-section of the thin yellow line in the Midbody image. Density indicates the images of the density of katanin p60 and of ß-tubulin (microtubules) in cross-section images. White arrowheads indicate the same position of images. Scale bars: 2 µm. Analyses were performed by confocal laser scanning fluorescence microscopy (FV-1000D; Olympus). C. Micrographs around the contractile ring. 3Y1 cells labeled for katanin p60 (green), actin (red), and DNA (blue). Merge indicates merged images of katanin p60, actin, and DNA, showing the localization of katanin p60 and the contractile ring at telophase. Cross-section analysis of the region between white arrowheads is shown in the Cross-section row. Scale bars: 5 µm (Horizontal) and 2.5 µm (Cross-section). Analyses were performed by confocal laser scanning fluorescence microscopy (FV-1000D; Olympus).

Katanin p60 localization was determined during mitosis by immunofluorescence analysis. From prophase to metaphase, katanin p60 was localized at the spindle pole in agreement with previous studies in other species (Figure 2A Pro and Meta) [25]. At anaphase, katanin localization at the spindle pole was reduced, but weak signals were newly detected around the cell equator (Figure 2A Ana). With the progression of cleavage furrow ingression, katanin p60 level decreased gradually at the spindle pole and clearly emerged on the central spindle (Figure 2A Telo). In cytokinesis, katanin p60 was localized around the terminal of the severed central spindle as a cap (Figure 2A Cytokinesis). These observations suggested that the function of katanin p60 is related to microtubule structure at the midbody during cytokinesis.

### Katanin p60 is distributed in a ring shape at the central spindle without katanin p80

To examine the function of katanin p60 at the midbody, its distribution at the midbody was examined in detail (Figure 2A Telo) (Figure 2 B Central spindle). The vertical (Z-axis) distribution of telophase katanin p60 at the central spindle had a ring-like shape (Figure 2 B Katanin p60) with little distribution within the inner area of this ring distribution. Microtubules in the same region partly overlapped and were localized inside the ring of katanin p60 (Figure 2B β-tubulin). Moreover, the katanin p60 ring distribution was localized mostly inside the contractile ring and partly colocalized with the inner portion of the actin ring distribution (Figure 2C). These results suggested that katanin p60 has a ring-like distribution in the gap between the inside of the contractile ring and the outside of the central spindle bundle at telophase. Around completion of cytokinesis, katanin p60 was localized specifically on and around (wrapping) the regions of packed central spindle microtubules flanking the narrow gap between two daughter cells, coincident with that of the midbody, which is called the Flemming body (Figure 2A Cytokinesis). These observations suggested that katanin p60 assembled as a covering on the central spindle inside the cell cortex around the contractile ring, and contraction of katanin p60 may destabilize the central spindle in the region of overlap with microtubules.

**Figure 3:**
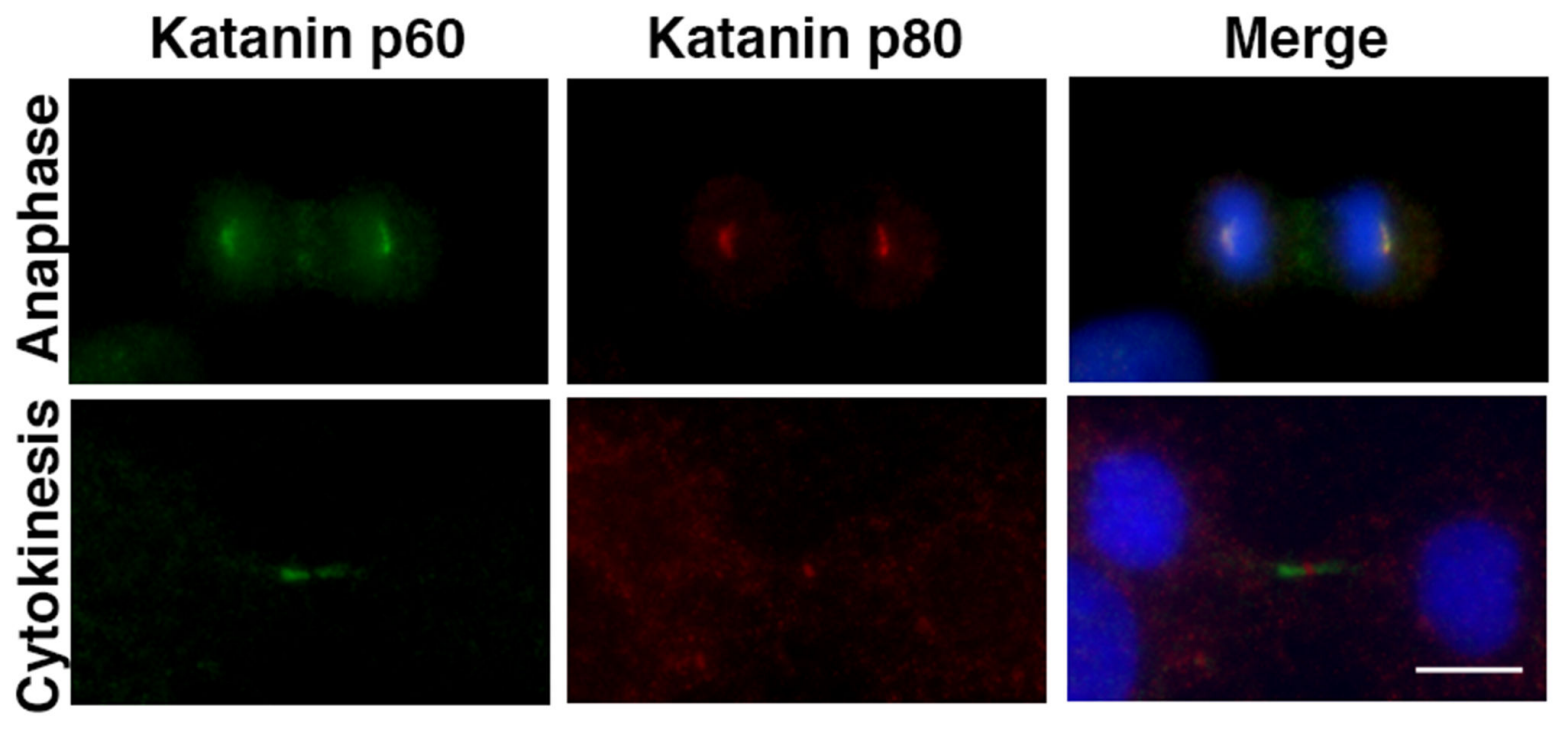
Katanin p80 subcellular localization was different from katanin p60 at the midbody. 3Y1 cells were labeled for katanin p60 (green), p80 (red), and DNA (blue). Merge indicates merged images of katanin p60, p80, and DNA, showing the localization at anaphase (Anaphase) and in cytokinesis (Cytokinesis) during mitosis. Katanin p60 localization at both the midzone and midbody during cytokinesis differed from katanin p80. Scale bars: 10 µm. Samples were fixed in methanol and analyzed by fluorescence microscopy (Axioskop II; Carl Zeiss).

Katanin p60 has been suggested to function as a severing enzyme, katanin, consisting of a heterodimer with katanin p80 subunit [24]. A recent study suggested that katanin p80 is localized at the midbody (especially at the Flemming body) with the candidate tumor suppressor LAPSER1 [28,29], and this localization was different from that of katanin p60 seen at the midbody in the present study. First, we performed immunostaining for katanin p80, and the results showed that katanin p80 localization matched that reported previously [28,29] (Figure S4). Therefore, we confirmed katanin p60 and p80 localization at the midbody by co-immunostaining (Figure 3). Katanin p60 was localized at both the spindle poles and midzone at anaphase (Figure 3 Katanin p60 and Anaphase), while katanin p80 was detected only at the spindle poles (Figure 3 Katanin p80), colocalizing with p60. There was no p80 signal at the midzone, in contrast to the localization of p60 (Figure 3 Merge). At the end of cytokinesis, katanin p60 was localized only at the regions of midbody microtubules flanking the Flemming body, while katanin p80 was localized specifically on the Flemming body where p60 was absent (Figure 3 Cytokinesis). These results suggested that katanin p60 functions independently of katanin p80 during cytokinesis at the midbody.

### Katanin p60 localization in the central spindle is not dependent on contractile ring activity

**Figure 4:**
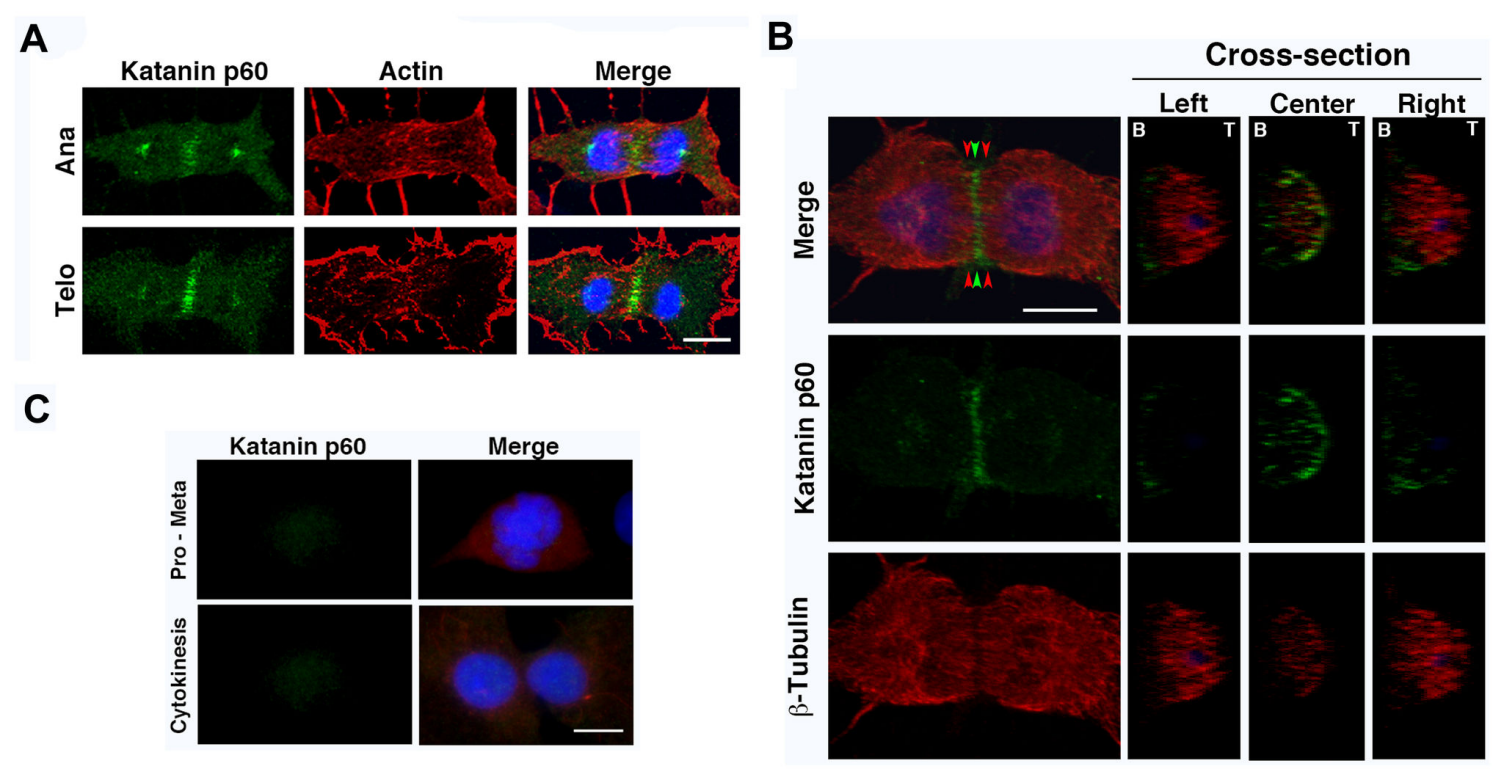
Katanin p60 was bundled with microtubules by contractility of the contractile ring and distributed in a microtubule-dependent manner. A. 3Y1 cells were treated with 10 µM blebbistatin for 1 h and then labeled for katanin p60 (green), actin (red), and DNA (blue). Merge indicates merged images of katanin p60, actin, and DNA, showing the localization of katanin p60, microtubules, and the contractile ring at anaphase (Ana) and telophase (Telo) during mitosis. Scale bars: 10 µm. Samples were fixed in methanol and analyzed by confocal laser scanning fluorescence microscopy (FV-1000D; Olympus). B. 3Y1 cells were treated with 10 µM blebbistatin for 1 h and then labeled for katanin p60 (green), β-tubulin (red), and DNA (blue). Merge indicates merged images of katanin p60, β-tubulin and DNA, showing the localization of katanin p60 and microtubules at telophase (Telo) during mitosis. Cross-section showing vertical images of the distributions of katanin p60 (green), β-tubulin (red), and DNA (blue). Green arrowheads indicate vertical analysis point corresponding to the “Center” of the plane where katanin p60 was present. Red arrowheads indicate vertical analyses points corresponding to “Right” and “Left” at both sides neighboring the plane where katanin p60 was present. “B” and “T” indicate “Bottom” and “Top” of the cell, respectively. Scale bars: 10 µm. Samples were fixed in methanol and analyzed by confocal laser scanning fluorescence microscopy (FV-1000D; Olympus). C. 3Y1 cells were treated with 10 µM nocodazole for 30 min, and then labeled for katanin p60 (green), β-tubulin (red), and DNA (blue). Merge indicates merged images of katanin p60, β-tubulin, and DNA at prophase – metaphase (Pro – Meta) and at cytokinesis (Cytokinesis). Both katanin p60 and microtubules disappeared. Scale bars: 10 µm. Samples were fixed in methanol and analyzed by fluorescence microscopy (Axioskop II; Carl Zeiss).

The relationship between the distributions of the contractile ring activity and katanin p60 during cytokinesis was examined (Figure 4). We used the myosin II ATPase inhibitor blebbistatin [33] to analyze the relationships among katanin p60, microtubules, and contractile ring activity. Following treatment with blebbistatin, the contractile ring disappeared completely during cytokinesis, indicating the proper effect of blebbistatin (from anaphase to telophase; Figure 4A Ana and Telo). The cell cycle phase (especially from anaphase to cytokinesis) of each cell was determined both by the state of nuclear DNA and the ratio of katanin distribution between the spindle pole and central spindle (refer to Figure 2 Ana – Cytokinesis). Katanin p60 distribution was concentrated on short fragments in a parallel manner over the cell equator in blebbistatin-treated cells (Figure 4A). Moreover, katanin p60 localization matched that of microtubules within the central spindle at telophase in blebbistatin-treated cells (Figure 4 B). Central spindle density in the area of katanin p60 localization was lower than the surrounding microtubule density (Figure 4B Microtubule). These results indicated that the central spindle and katanin p60 were bundled by myosin II contraction activity, and katanin p60 acts on the central spindle. Analyses of cross-sections indicated that katanin p60 was localized mainly in the cortical region of the cleavage furrow in a broken ring-like pattern, and showed a low density in the inner region (Figure 4B Cross-section). This distribution was consistent with the results shown in Figure 2B. Microtubules were evenly distributed at low density inside the broken ring of katanin p60, and the outer boundary of microtubule distribution was restricted to the inner edge of the katanin p60 broken ring (Figure 4B Center). Cross-sections adjacent to the region of katanin localization (Left and Right) showed that microtubules were distributed evenly at high density to the cortical region without katanin p60, and the region of low katanin p60 localization matched that in which microtubules were sparse (Figure 4B Left and Right). It is likely that katanin p60 destabilized central spindle microtubules at the midzone. However, it was reported previously that centralspindlin accumulated on the central spindle overlap region and blocked access to factors such as anti-tubulin antibody, and therefore the central spindle often showed a gap without staining when cells were labeled with anti-tubulin antibody [34,35]. Therefore, the contribution of katanin p60 catalytic activity to formation of the microtubule gap in the central spindle remains unclear.

Next, the relationship between katanin p60 localization at the midbody and central spindle microtubules was examined (Figure 4C). On treatment with nocodazole, microtubules were completely disassembled (Figure 4C Merge), and no katanin p60 localization was seen (Figure 4C Katanin p60). These results indicated that katanin p60 accumulated on the central spindle dependent only on the microtubule structure and was bundled by contraction of actomyosin purse strings. 

### Katanin p60 destabilizes microtubules at the central spindle and the midbody

**Figure 5:**
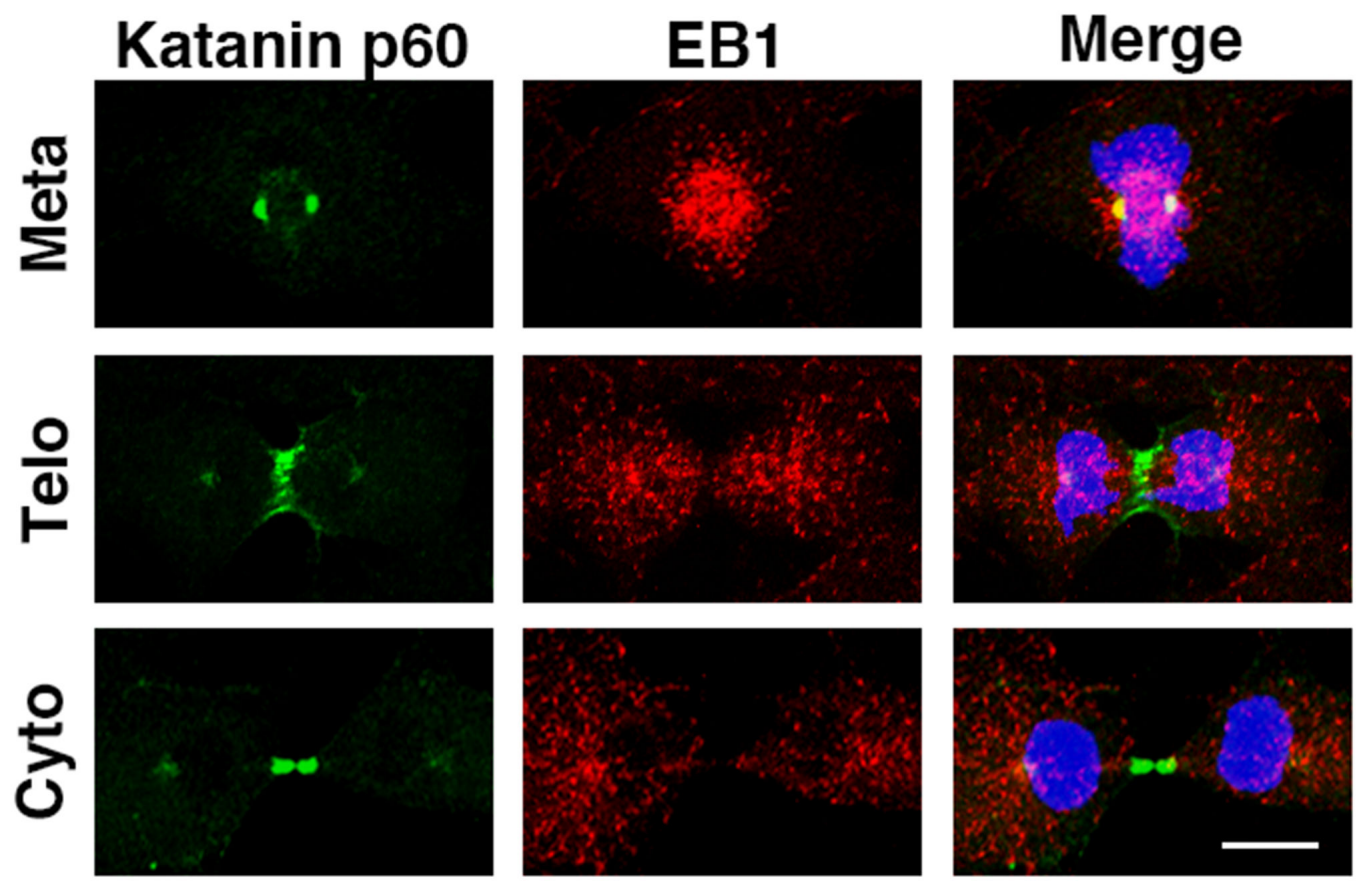
Katanin p60 localization was restricted in the area of non-growing microtubules during cytokinesis. 3Y1 cells labeled for katanin p60 (green), EB1 (red) (EB1), and DNA (blue). Merge indicates merged images of katanin p60, EB1, and DNA, showing the localization of katanin p60 at metaphase (Meta), telophase (Telo), and cytokinesis (Cyto) during mitosis. Scale bars: 10 µm. Samples were fixed in methanol and analyzed by confocal laser scanning fluorescence microscopy (FV-1000D; Olympus).

To investigate the function of katanin p60 at the midbody, we investigated the relationship between microtubule activity/structure and katanin p60 localization. We examined the localizations of both katanin p60 and EB1, which binds the ends of growing microtubules (Figure 5, anti-EB1 antibody reactivity was checked in Figure S1; see Materials & Methods). EB1 had a comet-like appearance on the ends of microtubules in the cytoplasm throughout the cell cycle, as reported previously (Figure S1) [36]. EB1 localization was especially dense in the spindle region and katanin p60 was colocalized with EB1 at metaphase (Figure 5 Meta). Moreover, EB1 did not localize in part of the central spindle at telophase (Figure 5 Telo) or in part of the midbody at the end of cytokinesis (Figure 5 Cyto), and was more restricted in the area without katanin p60 (Figure 5 EB1). However, microtubules were observed in areas with both katanin p60 and EB1 localization (Figures 1 and 2). These observations indicated that the presence of katanin p60 at the central spindle and the midbody was inversely correlated with EB1 localization, and microtubules plus ends at the central spindle and the midbody with katanin p60 did not polymerize. Thus, katanin p60 may destabilize microtubules and facilitate microtubule depolymerization during cytokinesis.

**Figure 6:**
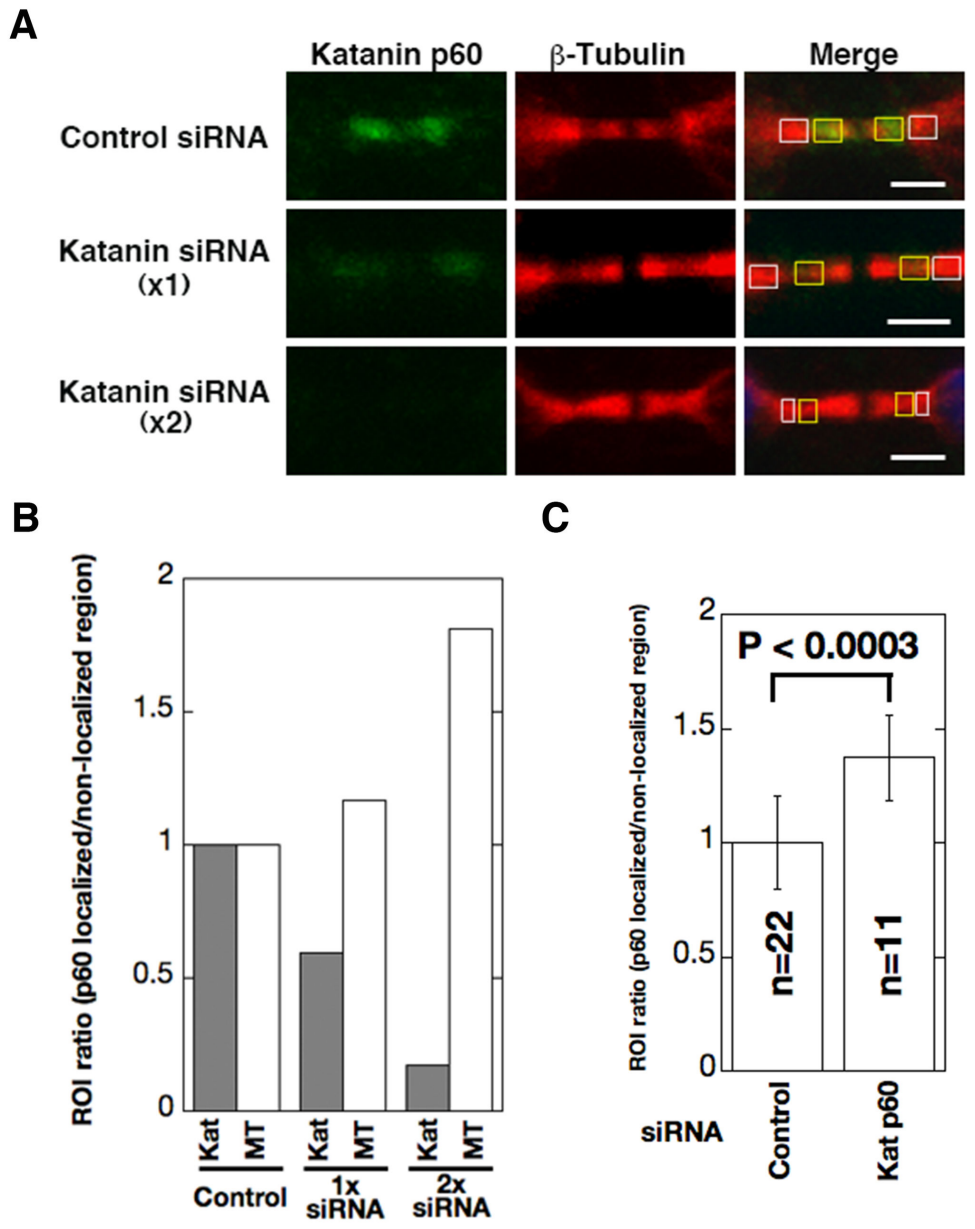
Katanin p60 destabilized midbody microtubules and affected cytokinesis in mitosis. A. 3Y1 cells were transfected with control siRNA (Control siRNA) or either once or twice with katanin p60 siRNA (Katanin siRNA (1×) and Katanin siRNA (2×), respectively). Forty-eight hours after transfection, cells were labeled for katanin p60 (green) and β-tubulin (red). Merge indicates merged images of katanin p60 and ß-tubulin, showing the localization of katanin p60 and β-tubulin (microtubules) on the midbody at cytokinesis. Yellow and white boxes in merged images (Merge) indicate katanin p60 localized and absent regions, respectively. Scale bars: 2 µm. Samples were fixed in methanol and analyzed by confocal laser scanning fluorescence microscopy (FV-1000D; Olympus). B. Measured ROI value ratios were calculated from the fluorescence intensities of the target regions. Calculations were performed as follows for each merged image in Figure 6A: ROI value of katanin p60 localized region (yellow boxes)/ROI value of katanin p60 absent region (white boxes). Gray and open boxes indicate relative ROI value ratio of katanin p60 and microtubules, respectively. ROI value ratio of control siRNA-treated cells was fixed to 1. C. Measured ROI value ratio analyses with the same method as described in Figure 6B were performed against cells treated with control siRNA (*n* = 22) and katanin p60 siRNA (*n* = 11) in four independent transfection experiments. Obtained values against microtubule ROI values were compared by *t* test.

To examine the relationship between microtubule destabilization and katanin p60 localization during cytokinesis, we examined whether katanin p60 localization affected the density of microtubules in the midbody area. We performed siRNA experiments and measured microtubules and katanin p60 density in the midbody (Figure 6 A). As shown in Figure 1C, midbody katanin p60 was partly repressed by single siRNA treatment and completely by double siRNA treatment. We performed immunostaining of siRNA-treated cells (control siRNA, 1×, and 2× katanin siRNA treatment), and measured and compared katanin density and microtubules on katanin localized (yellow squares) and absent regions (white squares) (Figure 6A Merge). The results are shown in Figure 6B as katanin and microtubule intensities (ratio of katanin localized region/absent region) relative to control siRNA-treated cells. Katanin p60 intensity was decreased by siRNA treatment (Fig. 6B gray boxes) and microtubule intensity (white boxes) increased in inverse proportion to the decrease in katanin level (Figure 6B). Next, we performed immunostaining and measurement of siRNA-treated cells according to the same method as described in Figure 6A in four independent experiments (Figure 6C). Microtubule density in the katanin p60 localized region of katanin p60 siRNA-treated cells was significantly greater than that of control siRNA-treated cells (*P* < 0.0003, Figure 6C). These results strongly suggested that katanin p60 prohibited microtubule elongation and destabilized midbody microtubules during cytokinesis.

### Katanin p60 contributes to accurate cytokinesis

**Figure 7:**
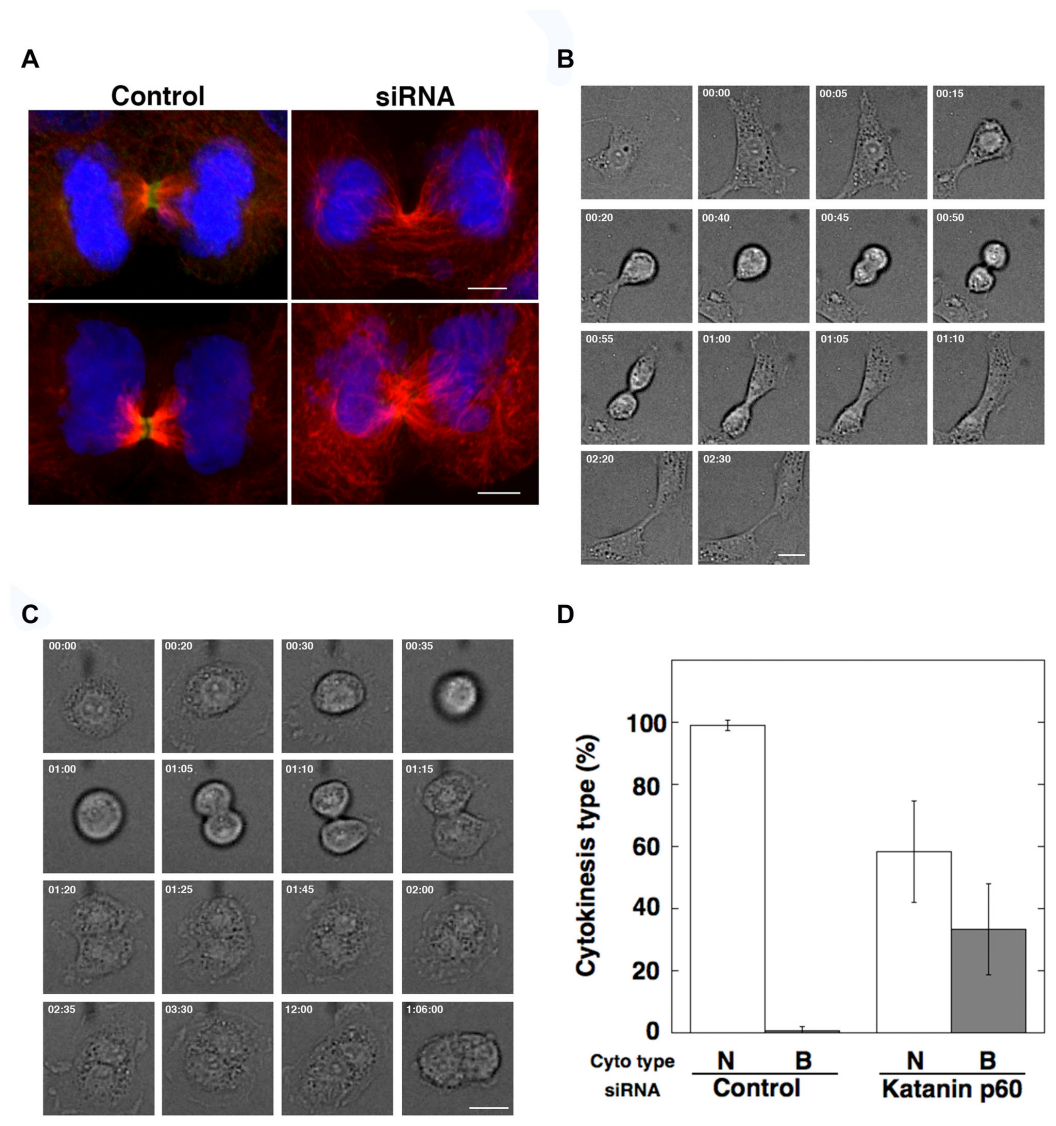
Katanin p60 inhibition leads to incomplete cytokinesis. A. 3Y1 cells labeled for katanin p60 (green), microtubules (red), and DNA (blue). Control and siRNA indicate non-treated 3Y1 cells and 3Y1 cells transfected with katanin p60 siRNA, respectively. Scale bars: 5 µm. Samples were fixed in methanol and analyzed by confocal laser scanning fluorescence microscopy (FV-1000D; Olympus). B and C. Time courses of differential interference contrast (DIC) microscopy images at mitosis of control siRNA-treated cell (B) and katanin p60 siRNA-treated cell (C). Scale bar: 20 µm. D. Types of cytokinesis of siRNA-treated cells are shown. Open and shaded boxes indicate normal cytokinesis completion (N) and incomplete cytokinesis by regression leading to binucleate cell formation (B), respectively. Averages and SD were calculated from three independent experiments (total 13 and 15 independent observation fields of control and katanin p60 siRNA treatment, respectively). Control: control siRNA-treated cell; Katanin p60: katanin p60 siRNA-treated cell.

As described above, katanin p60 was localized at the midbody and controlled microtubule stability. However, it was unclear whether katanin p60 severed microtubules during telophase because the central spindle was often found to be inaccessible to the antibody due to centralspindlin binding, as shown in Figure 4B. Therefore, we performed RNAi experiments with a higher concentration of siRNA (5× the volume used in Figure 6) to markedly repress katanin p60 in telophase cells. We observed sufficient repression of katanin p60 in telophase cells (Figure 7A, green). In the control cells at telophase, katanin p60 was localized to a narrow region of the central spindle (Figure 7A, Control). Microtubule density was lower in the region of katanin p60 localization than in the adjacent region (Control, upper), and the central spindle seemed to be separated into two parts with a gap showing no staining, and each end of stained microtubule bundle was likely to be covered independently with a katanin p60 cap (Control, lower). These results suggested that katanin p60 destabilized microtubules in the central region. Alternatively, it is possible that microtubules were not stained in central region because of antibody exclusion in the central region of the intact midzone due to high-density accumulation of central spindle-binding factors. In siRNA-treated telophase cells, microtubules were denser and longer than in the control cells and there was no gap in staining in the equatorial plane (Figure 7A siRNA). These results indicated that katanin p60 repression was correlated with increased microtubule organization and with the disappearance of the region without microtubule staining at the central spindle. Therefore, combined with the results shown in Figure 6 and the catalytic characteristics of katanin p60, these observations suggested that katanin p60 was localized on and destabilized the central spindle during the process of cytokinesis.

**Figure 8:**
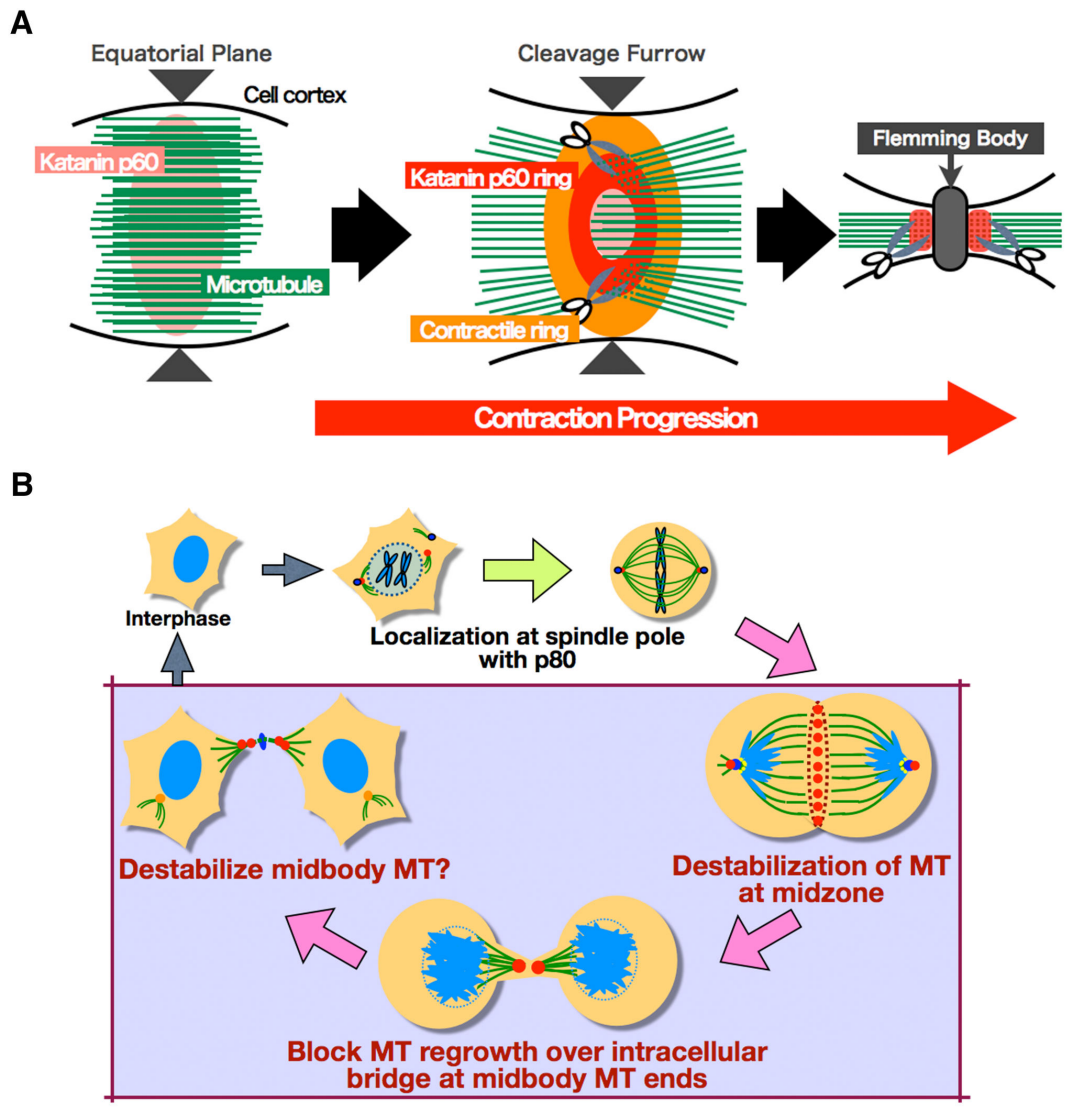
Model for Katanin p60 function during cytokinesis. A. Model of katanin p60 function at the midbody. B. Katanin p60 function through the cell cycle.

If katanin p60 has a functional role in regulating cytokinesis, depletion of katanin p60 activity may perturb the cell cycle. Time-lapse analyses indicated that the growth rate of siRNA-treated cells was markedly reduced. The specific growth rate of siRNA cells was -0.034 ± 0.012 h^-1^, while that of control cells was 0.756 ± 0.14 h^-1^. RNA interference of katanin p60 markedly inhibited mitosis entry and induced apoptosis more than mitosis entry, so the growth rate of siRNA-treated cells was < 0. Phenotypically, siRNA-treated interphase cells showed significant enlargement (Figure S5), indicating that inhibition of mitosis entry by katanin p60 depletion induced accumulation of G2 cells containing the cellular materials of two daughter cells. However, a small proportion of katanin p60 siRNA-treated cells entered mitosis. Therefore, we analyzed the fate of mitotic cells by time-lapse analyses of siRNA-treated cells. Cytokinesis of control cells showed normal ingression of the cytokinetic furrow followed by abscission, and the total mitotic process completed within about 2 h, corresponding to the time course of normal mitosis (Figure 7B). Katanin p60 siRNA-treated cells frequently showed cytokinetic failure (Figure 7C). Cells entered mitosis at time 30 min, cleavage furrow ingression was observed at time 1 h 5 min, regression started at 1 h 15 min, binucleate cells emerged with disappearance of the septum between daughter cells at 1 h 25 min, and binucleate cells shrunk and lost movement around 30 h (1:06:00). Finally, binucleate cells underwent apoptosis (Video S2). Therefore, we analyzed the probability of cytokinetic failure of katanin p60 siRNA-treated cells by defining the cytokinetic process of all cells entering mitosis by time-lapse observation. Normal cytokinesis was observed at a rate of only 56% (control siRNA-treated cells 99.1%) and the rate of binucleate cell production by cytokinesis failure was 39% of mitoses (control siRNA-treated cells 0.9%, remaining 5% was abnormal cell division that reached apoptosis) (Figure 7D). These results indicated that inhibition of katanin p60 brought about 43-fold cytokinetic failure. Similar results were obtained by counting binucleate siRNA-treated cells (Figure S6). The frequency of binucleate cells in the total cell population was especially high (9.9-fold compared to controls; 783 siRNA cells and 1804 control cells tested, respectively) at 48 h after transfection and decreased gradually thereafter but remained higher than that of controls. These results suggested that inhibition of katanin p60 activity induced cytokinetic failure by inhibition of cleavage furrow ingression. Finally, the results of this study revealed the midzone – midbody localization and function of katanin p60 during cytokinesis (Figure 8). Katanin p60 is an important regulator of central spindle dynamics.

## Discussion

The results of the present study demonstrated the cellular localization of katanin p60 and its function in cytokinesis. Katanin p60 not in complex with katanin p80 subunit showed a ring-shaped distribution in the gap between the inside of the contractile ring and the central spindle bundle (Figure 3). When the contractile ring was disrupted by blebbistatin (Figure 4), the ring-shaped distribution of katanin p60 disappeared but localization of katanin p60 on the microtubules in the plane of the cell equator remained. These results indicated the following mechanism for formation of the ring-shaped katanin p60 distribution. Katanin p60 accumulates around the cortical part of the cytosol at the cell equator in late anaphase. The mechanism underlying this accumulation is still unknown, but it is likely similar to the accumulation of building blocks of central spindle-binding factors, such as centralspindlin, at the equatorial plane. When katanin p60 accumulates in the equatorial plane, the central spindle is covered with central spindle-binding proteins, and it is difficult for katanin p60 to gain access to the microtubules. After accumulation of katanin p60 in the equatorial plane (perhaps uniformly), the contractile ring then pushes katanin p60 and the central spindle inward by virtue of contraction. As a result, katanin p60 is forced to make tight contact with the central spindle bundle, destabilizes the microtubules, and then combines with katanin p60 in the constricted area, and is finally likely to show dense accumulation at the outer area of the equatorial plane (Figures 2B, C and 4B). Although it remains unclear whether katanin p60 binds to microtubules directly or via central spindle-binding factors, such as centralspindlin [34,35], PRC1 [3,37], etc., at telophase (Figure 8), our results suggested that katanin p60 acts on the central spindle via binding with central spindle-binding factors (Figures 2 and 4B). 

On the central spindle, augmin was reported to induce microtubule generation in a microtubule-dependent manner [15-17]; however, the mechanism of depolymerization has not been clarified. The results of the present study indicated that katanin p60 functions as a destabilizing factor of the central spindle at the midbody. Augmin is a positive regulator for generation of the central spindle, and katanin p60 is a negative regulator for destabilization of the central spindle in the region of actomyosin purse string contraction and prevents re-elongation of the divided central spindle. Augmin deficiency induces central spindle abnormalities and results in the generation of multinucleated cells [38,39]. Katanin p60 repression enhanced microtubule density at midbody by stabilizing the central spindle, caused incomplete cleavage furrow ingression, and finally induced binucleate cell formation by cytokinetic failure. These observations strongly suggested that regulation of central spindle stability is very important for appropriate completion of cytokinesis.

Our RNAi experiments showed that katanin p60 plays an important role in mitosis entry and cleavage furrow ingression. The detailed functions of katanin on the spindle pole have been confirmed [18,26,40-42]. The importance of katanin p60 at the early stage of cytokinesis was demonstrated by RNAi study in bloodstream-stage *Trypanosoma brucei* [22]. Moreover, localization of katanin at the midbody was observed in HeLa cells [27]. These observations suggested that katanin localization and function in cytokinesis are ubiquitous and important among animal cells.

The structure of the N-terminal region of katanin p60 was reported to resemble that of Vsp4, which is a component of the ESCRT [30]. Moreover, Morita et al. reported that human ESCRT-III and VPS4 proteins are required for centrosome and spindle maintenance [31]. These studies suggested that there are functional and structural relationships between spindle and central spindle/midbody, and katanin p60 may be intimately involved in both the spindle and central spindle function. Taken together with the findings of the present study, these observations indicate that katanin is localized to the central spindle and the midbody not only to regulate microtubule stability but also to recruit the membrane for promotion of cytokinesis. Spastin, another AAA ATPase microtubule-severing protein, is localized predominantly at the midbody and interacts with CHMP1B, a component of the ESCRT machinery with roles in membrane cleavage, including cytokinesis [20,21]. These observations indicate the coupling of central spindle dynamics to membrane traffic of cytokinesis and suggest that katanin p60 may affect cytokinesis by regulating central spindle stability and membrane traffic.

It was reported that katanin p80 was localized to the Flemming body of the midbody at the end of cytokinesis [28,29]. We also confirmed these results (Figure 3), and this pattern of katanin p80 localization was not consistent with that of katanin p60 at the midzone and midbody. These results suggested that another binding partner(s) of katanin p60 was responsible for translocation to the midzone or that there may be a specific translocation signal (phosphorylation, neddylation, ubiquitination, *etc.*) for katanin p60 but not p80. At the Flemming body, katanin p80 was suggested to bind and interact with LAPSER1, a putative cytokinetic tumor suppressor, and to be involved in inhibition of microtubule severing [28,29]. Taken together with our results, these observations suggest that microtubule severing at the midzone and midbody during cytokinesis may be achieved by several mechanisms, including the activities of katanin p60, katanin p80, and LAPSER1.

The results of the present study indicated cell cycle-specific functions of katanin p60, especially in cytokinesis. Katanin p60 repression experiments by siRNA suggested that the stability of katanin p60 was different between the midbody and the spindle pole. Our findings indicated that katanin p60 formed a complex with p80 at the spindle pole but not at the central spindle and midbody. Cummings et al. reported that katanin p60 was degraded by the ubiquitin-proteasome system during mitosis in HeLa cells [27]. Moreover, mei1, a katanin p60 homolog in *Caenorhabditis elegans* required for meiotic spindle formation, was shown to function in meiosis and to be degraded by the ubiquitin system under regulation of nedd8 modification [43], and mei1 degradation facilitates the transition from meiosis to mitosis [43-46]. *C. elegans* katanin in the meiotic spindle functions to increase the number and density of non-centrosomal spindle microtubules. This mechanism occurs in the vertebrate mitotic spindle, although to a much lesser extent than in *C. elegans* meiosis [41]. These observations indicated that the stability of katanin p60 was different in relation to its binding partner that defined the affinity to protein degradation systems, such as the ubiquitin-proteasome system.

## Supporting Information

Figure S1 **Specificity of mouse anti-EB1 monoclonal antibody.** Mouse anti-EB1 monoclonal antibody (Red) was used for immunofluorescence analyses with rat anti-α-tubulin monoclonal antibody (Green) on 3Y1 cells. EB1 was localized specifically on the ends of microtubules. The white box indicates expanded analyses area. Scale bar: 10 µm.
(TIF)

Figure S2 **Specificity of affinity-purified anti-katanin p60 antibody.** A. WB, Western blotting analysis of 3Y1 cell extract with affinity-purified anti-katanin p60 antibody. CBB, Coomassie brilliant blue-stained image of 3Y1 cell extract. 3Y1 cell extract was prepared as follows. 3Y1 cells grown in dishes 6 cm in diameter (TPP) were harvested, washed twice with PBS, and the cell pellet was lysed with the same volume of 2× SDS sample buffer (125 mM Tris-HCl, pH 6.8, 20% glycerol, 4% SDS, 288 mM 2-mercaptoethanol, 10 µg/mL of bromophenol blue). The same amounts of extract (10 µg) were used for Western blotting and CBB analyses. B. WB, Western blotting analysis of 1 ng of recombinant rat katanin p60 and rat KATNAL1 proteins with affinity-purified anti-katanin p60 antibody. CBB, Coomassie brilliant blue-stained image of 100 ng of both rat katanin p60 and KATNAL1 proteins.
(TIF)

Figure S3 **3Y1 cells labeled for katanin p60 (green), β-tubulin (red) and DNA (blue).** Merge indicates merged images of katanin p60, β-tubulin, and DNA showing the localization of katanin p60 at the spindle pole (Metaphase) and midbody (Cytokinesis) at mitosis. Anti-katanin p60 antibody was absorbed by purified recombinant katanin p60 protein before labeling (Absorption). Control indicates the images of 3Y1 cells labeled in the same manner as in Figure 1A. Scale bars: 10 µm. Samples were fixed in methanol and analyzed by fluorescence microscopy (Axioskop II; Carl Zeiss).
(TIF)

Figure S4 **Katanin p80 distributions during mitosis.** 3Y1 cells were labeled for katanin p80 (green), microtubules (red), and DNA (blue). Merge indicates merged images of katanin p60, microtubules, and DNA, showing the localization of katanin p80 during mitosis. Pro, Meta, Ana, and Cyto indicate images of prophase, metaphase, anaphase, and cytokinesis, respectively. Scale bars: 10 µm. Samples were fixed in methanol and analyzed by confocal laser scanning fluorescence microscopy (FV-1000D; Olympus).
(TIF)

Figure S5 **Katanin p60 siRNA treatment induced enlargement of cell size.** 3Y1 cells were treated with no siRNA, control siRNA, or with katanin p60 siRNA. Forty-eight hours after transfection, cells were labeled for β-tubulin (red) and DNA (blue), and the lengths of both the long and short axes (at right angles) of cells with similar nuclear size were determined. The product of both lengths was calculated and the mean and standard deviation were calculated from respective siRNA-treated cells (*n* = 159 for no siRNA, 137 for control siRNA, and 134 for katanin p60 siRNA). The *t* test was used for statistical analysis (*P* < 0.002).
(TIF)

Figure S6 **A**. **Time course of changes in the ratio of cytokinetic cells among mitotic cells**. The ratio of telophase cells was determined from the ratio of (number of telophase cells)/(number of mitotic cells) between cells treated with katanin p60 siRNA and control siRNA. B. Time course of changes in the ratio of binucleate cells. The ratio of binucleate cells was determined from the ratio of (number of binucleate cells)/(number of total cells inspected) between cells treated with katanin p60 siRNA and control siRNA. The value of control siRNA-treated cells was set to 1. Experiments were performed in triplicate. Error bars indicate standard error among experiments.
(TIF)

Video S1 **Control siRNA treated rat 3Y1 cells.**
(ZIP)

Video S2 **Katanin p60 siena treated rat 3Y1 cells.**
(ZIP)
