# Supplementary figures and images for: Katanin p60 Contributes to Microtubule Instability around the Midbody and Facilitates Cytokinesis in Rat Cells
Source: PLoS One. 2013 Nov 26;8(11):e80392. doi: 10.1371/journal.pone.0080392 (PMC3841192; doi:10.1371/journal.pone.0080392)

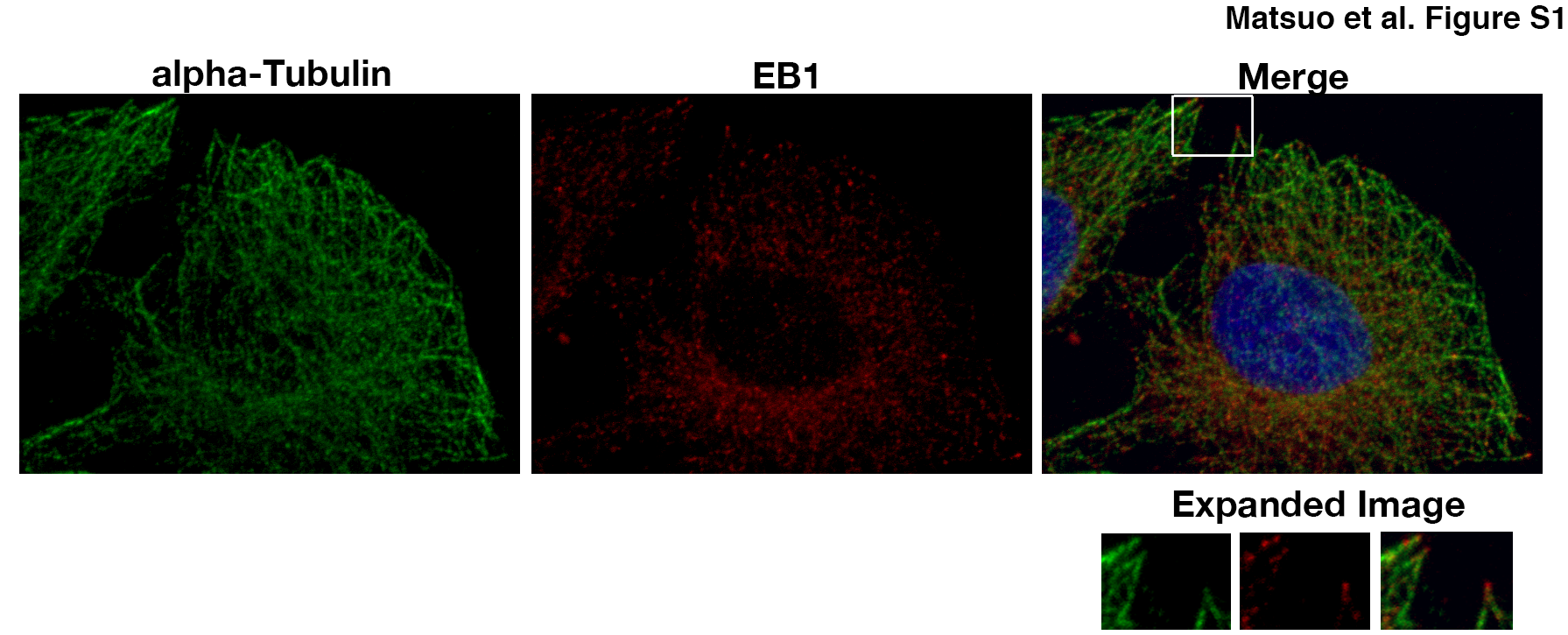

Supplement: Figure S1 — Specificity of mouse anti-EB1 monoclonal antibody. Mouse anti-EB1 monoclonal antibody (Red) was used for immunofluorescence analyses with rat anti-α-tubulin monoclonal antibody (Green) on 3Y1 cells. EB1 was localized specifically on the ends of microtubules. The white box indicates expanded analyses area. Scale bar: 10 µm. (TIF) [file pone.0080392.s001.tif]

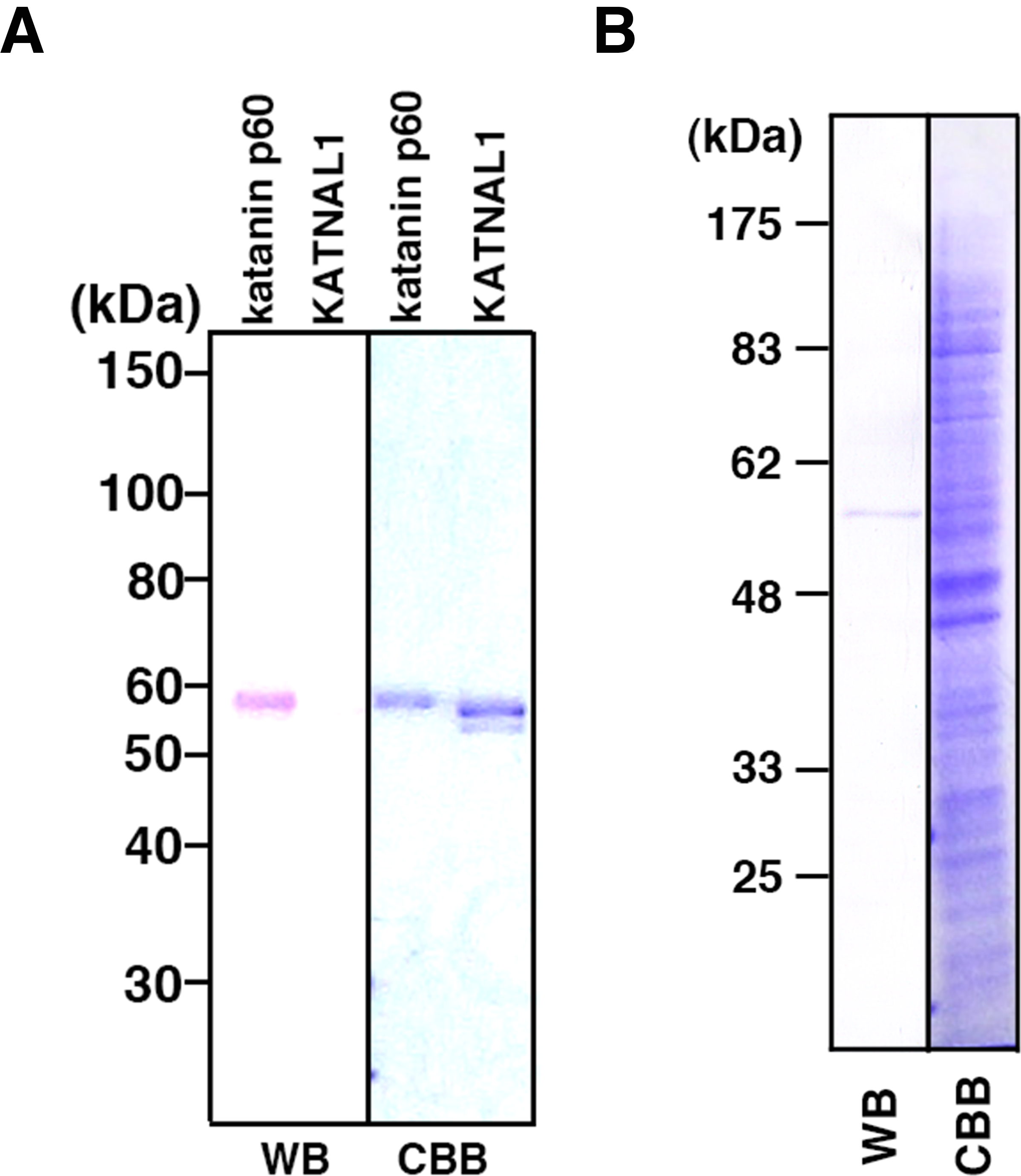

Supplement: Figure S2 — Specificity of affinity-purified anti-katanin p60 antibody. A. WB, Western blotting analysis of 3Y1 cell extract with affinity-purified anti-katanin p60 antibody. CBB, Coomassie brilliant blue-stained image of 3Y1 cell extract. 3Y1 cell extract was prepared as follows. 3Y1 cells grown in dishes 6 cm in diameter (TPP) were harvested, washed twice with PBS, and the cell pellet was lysed with the same volume of 2× SDS sample buffer (125 mM Tris-HCl, pH 6.8, 20% glycerol, 4% SDS, 288 mM 2-mercaptoethanol, 10 µg/mL of bromophenol blue). The same amounts of extract (10 µg) were used for Western blotting and CBB analyses. B. WB, Western blotting analysis of 1 ng of recombinant rat katanin p60 and rat KATNAL1 proteins with affinity-purified anti-katanin p60 antibody. CBB, Coomassie brilliant blue-stained image of 100 ng of both rat katanin p60 and KATNAL1 proteins. (TIF) [file pone.0080392.s002.tif]

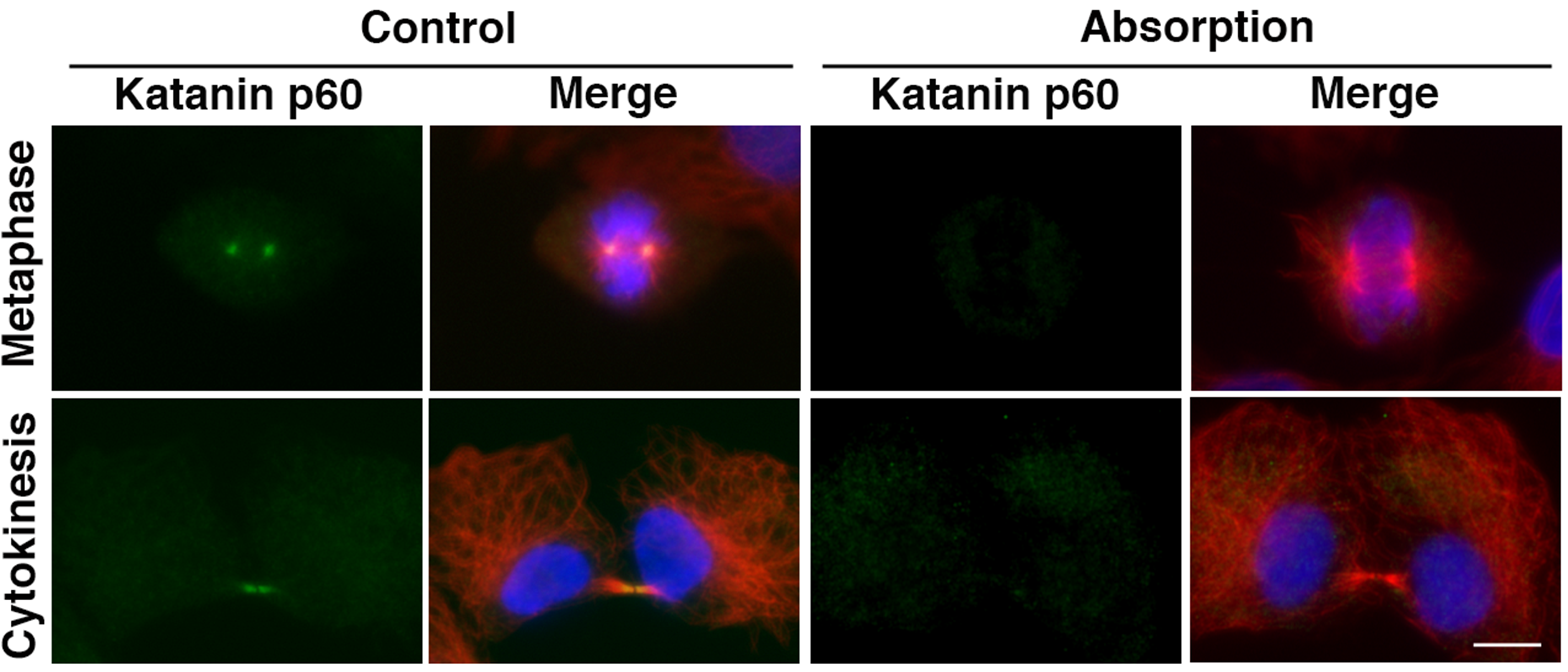

Supplement: Figure S3 — 3Y1 cells labeled for katanin p60 (green), β-tubulin (red) and DNA (blue). Merge indicates merged images of katanin p60, β-tubulin, and DNA showing the localization of katanin p60 at the spindle pole (Metaphase) and midbody (Cytokinesis) at mitosis. Anti-katanin p60 antibody was absorbed by purified recombinant katanin p60 protein before labeling (Absorption). Control indicates the images of 3Y1 cells labeled in the same manner as in Figure 1A. Scale bars: 10 µm. Samples were fixed in methanol and analyzed by fluorescence microscopy (Axioskop II; Carl Zeiss). (TIF) [file pone.0080392.s003.tif]

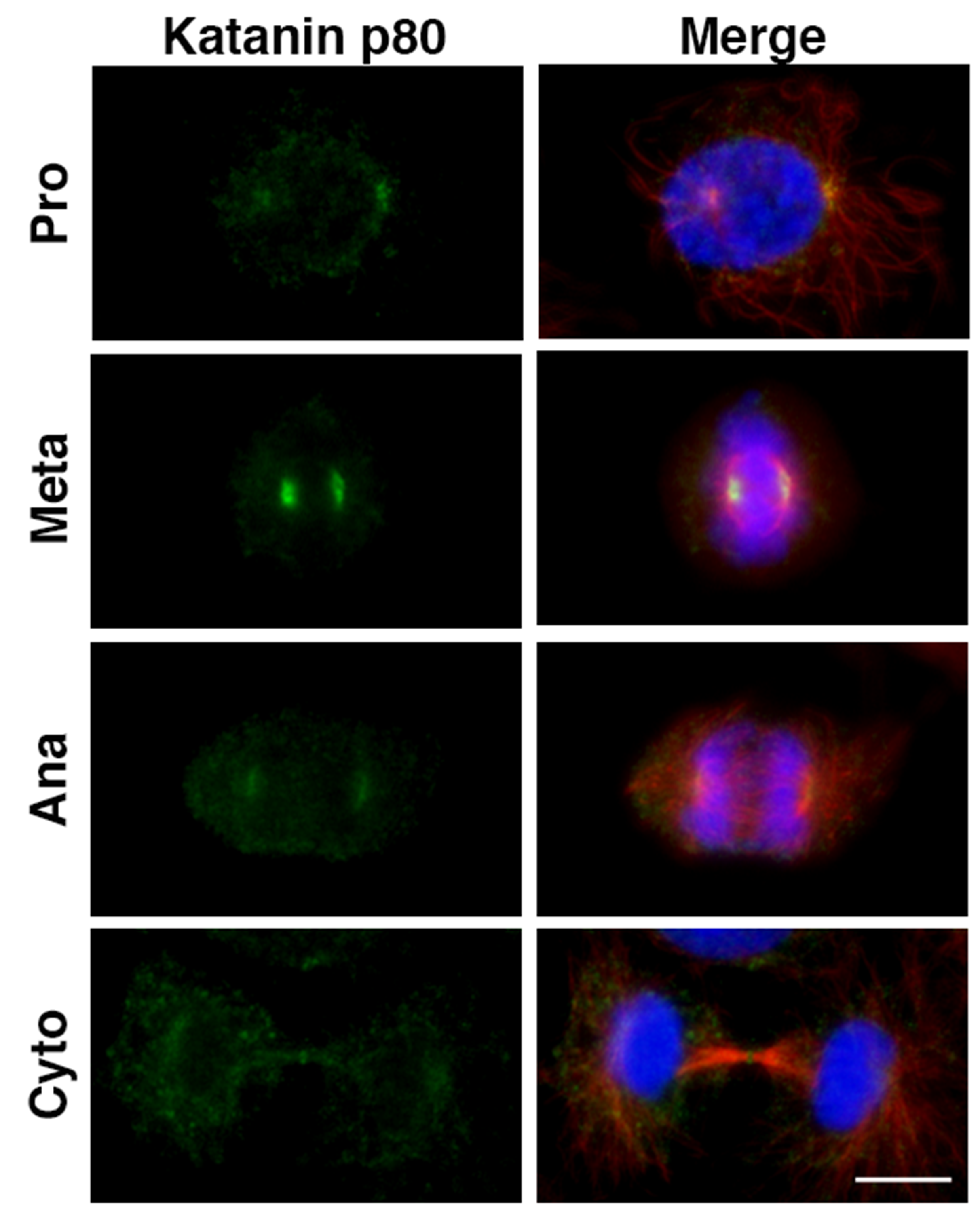

Supplement: Figure S4 — Katanin p80 distributions during mitosis. 3Y1 cells were labeled for katanin p80 (green), microtubules (red), and DNA (blue). Merge indicates merged images of katanin p60, microtubules, and DNA, showing the localization of katanin p80 during mitosis. Pro, Meta, Ana, and Cyto indicate images of prophase, metaphase, anaphase, and cytokinesis, respectively. Scale bars: 10 µm. Samples were fixed in methanol and analyzed by confocal laser scanning fluorescence microscopy (FV-1000D; Olympus). (TIF) [file pone.0080392.s004.tif]

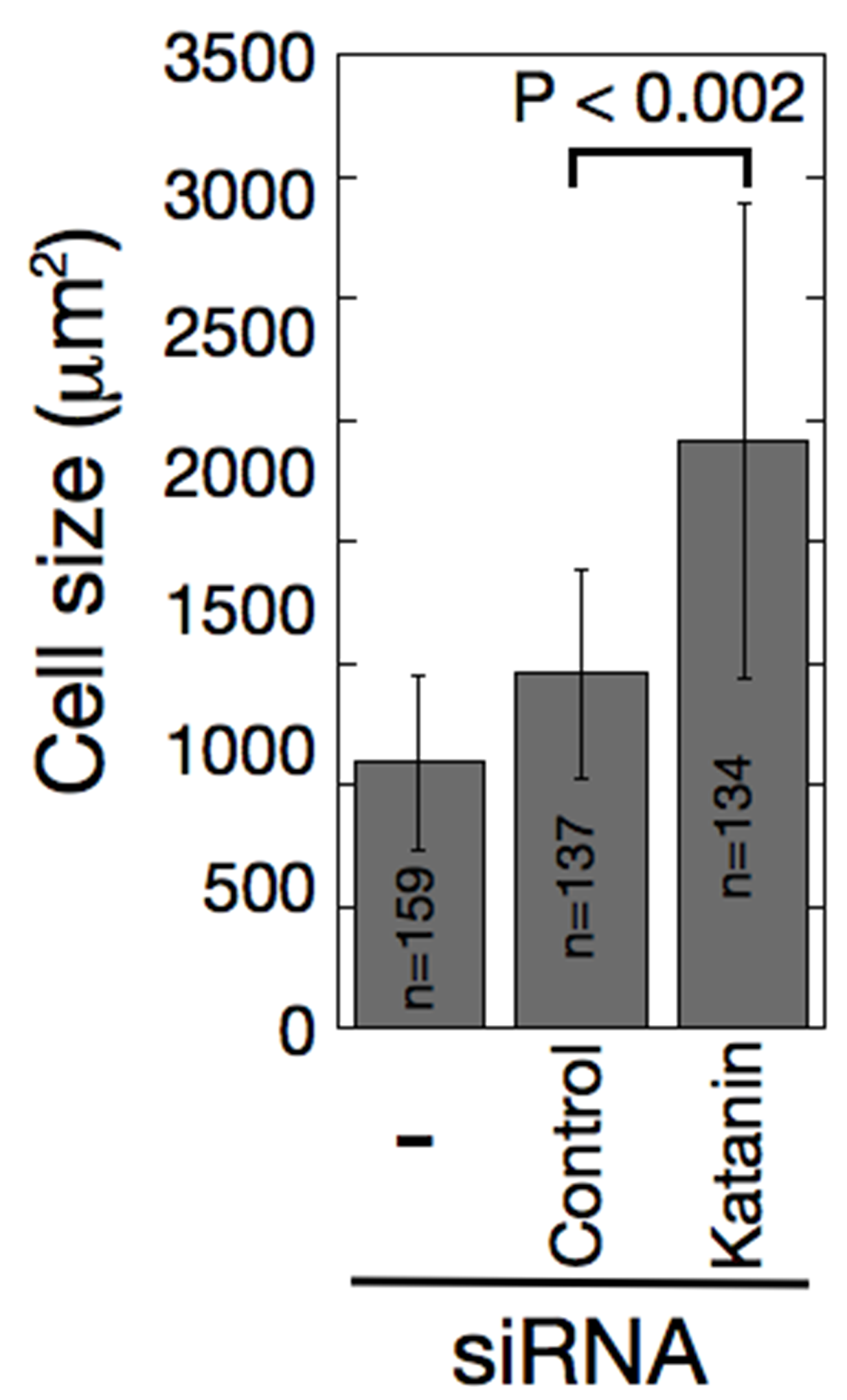

Supplement: Figure S5 — Katanin p60 siRNA treatment induced enlargement of cell size. 3Y1 cells were treated with no siRNA, control siRNA, or with katanin p60 siRNA. Forty-eight hours after transfection, cells were labeled for β-tubulin (red) and DNA (blue), and the lengths of both the long and short axes (at right angles) of cells with similar nuclear size were determined. The product of both lengths was calculated and the mean and standard deviation were calculated from respective siRNA-treated cells (n = 159 for no siRNA, 137 for control siRNA, and 134 for katanin p60 siRNA). The t test was used for statistical analysis (P < 0.002). (TIF) [file pone.0080392.s005.tif]

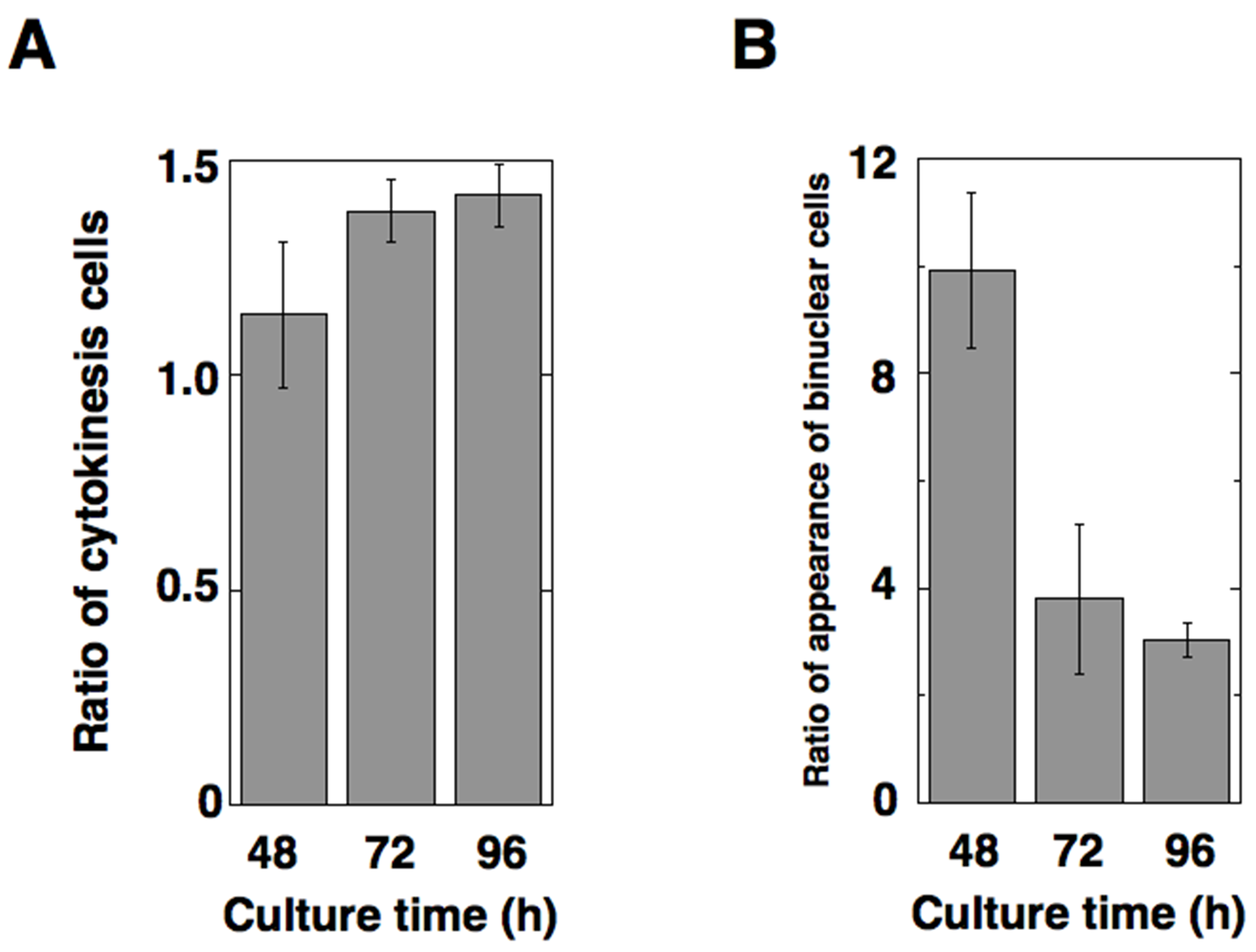

Supplement: Figure S6 — A. Time course of changes in the ratio of cytokinetic cells among mitotic cells. The ratio of telophase cells was determined from the ratio of (number of telophase cells)/(number of mitotic cells) between cells treated with katanin p60 siRNA and control siRNA. B. Time course of changes in the ratio of binucleate cells. The ratio of binucleate cells was determined from the ratio of (number of binucleate cells)/(number of total cells inspected) between cells treated with katanin p60 siRNA and control siRNA. The value of control siRNA-treated cells was set to 1. Experiments were performed in triplicate. Error bars indicate standard error among experiments. (TIF) [file pone.0080392.s006.tif]
